# Plasmonic Hot‐Carrier Engineering at Bimetallic Nanoparticle/Semiconductor Interfaces: A Computational Perspective

**DOI:** 10.1002/smll.202410173

**Published:** 2025-02-16

**Authors:** Mani Mani, Kevin Mariandry, Uma V. Ghorpade, Sankhadip Saha, Ravindra Kokate, Rishabh Mishra, Michael P. Nielsen, Richard Tilley, Bingqiao Xie, Mahesh P. Suryawanshi, Priyank V. Kumar

**Affiliations:** ^1^ School of Chemical Engineering UNSW Kensington NSW 2052 Australia; ^2^ School of Chemistry UNSW Kensington NSW 2052 Australia; ^3^ School of Photovoltaic and Renewable Energy Engineering UNSW Kensington NSW 2052 Australia; ^4^ Institute of Chemical Sciences and Engineering (ISIC) École Polytechnique Fédérale de Lausanne (EPFL) Lausanne 1015 Switzerland

**Keywords:** bimetallic, hot‐carrier transfer, interface, plasmonics, time‐dependent density functional theory

## Abstract

Plasmonic catalysis employs plasmonic metals such as Ag, Au, Cu, and Al, typically in combination with semiconductors, to drive diverse redox chemical reactions. These metals are good at harnessing sunlight, owing to their strong absorption cross‐sections and tunable absorption peaks within the visible range of the solar spectrum. Unfortunately, facilitating plasmon‐induced hot‐carrier separation and subsequently harvesting them to improve catalytic efficiencies has been a problem at monometallic particle‐semiconductor interfaces. To overcome this issue, this perspective focuses on recent computational methods and studies to discuss the advantages of designing bimetallic particles (core‐shell or core‐satellite), with a plasmonic‐metal core and a less‐plasmonic‐metal shell on top, and coupling them with semiconductors. The aim of this approach is to favorably modify the interface between the plasmonic‐metal particle and the semiconductor by introducing a thin section of a non‐plasmonic metal in between. This approach is expected to enhance hot‐carrier separation at the interface, preventing fast electron–hole recombination within the plasmonic‐metal particle. Through a careful design of such bimetal/semiconductor configurations, by varying the size and composition of the non‐plasmonic metal for example, and through appropriate utilization of quantum‐mechanical modeling and experimental techniques, it is anticipated that plasmonic hot‐carrier generation and separation processes can be studied and controlled in such systems, thereby enabling more‐efficient plasmonic devices.

## Graphical abstract

This perspective highlights the benefits of designing bimetallic particles (core‐shell or core‐satellite structures) with a plasmonic metal core and a less‐plasmonic metal shell, coupled with semiconductors, to address the challenge of plasmon‐induced hot‐carrier separation at monometallic particle‐semiconductor interfaces. The discussion is facilitated by recent quantum‐mechanical studies supported by experimental results. Further leveraging the discussed approaches could pave the way for a deeper understanding of the interfaces leading to more efficient plasmonic devices.

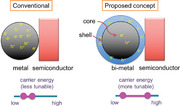

## 1 Introduction

Heterogeneous photocatalysis strives to harness sunlight and convert it into chemical energy.^[^ ^1^ ^]^ This process seeks to replicate natural photosynthesis, where plants convert sunlight into chemical energy. The field has traditionally relied on semiconductors to drive chemical reactions.^[^ ^2^ ^]^ When these semiconductors are exposed to light with energy greater than their band‐gap, they absorb the light and create electron‐hole pairs. These high‐energy charge carriers (generally referred to as hot electrons and hot holes) then separate and facilitate chemical transformations at catalytically active sites on the semiconductor's interface with reactants.

However, there are inherent limitations associated with such photocatalysts. For instance, materials like TiO_2_ have a relatively high bandgap, making them unable to utilize the visible part of sunlight for generating electron–hole pairs. They can only be excited by UV photons, which constitute a mere 5% of solar flux.^[^ ^3^ ^]^ Furthermore, the diffuse nature of solar flux presents challenges, with approximately 100 mW (or roughly 10^17^ solar photons) striking each square centimeter of surface per second. This means that, for a 2D surface, only about 100 solar photons interact with a single surface atomic site (typically covering an area of 10 Å^2^) each second.^[^ ^4^ ^]^ These limitations impose significant constraints on maximum reaction rates achievable.

To address these challenges, researchers have explored the use of semiconductor photocatalysts that can absorb visible light,^[^ ^3^, ^5^ ^]^ which is a distinct topic and will not be covered here. Another promising solution involves incorporating plasmonic monometallic nanoparticles (pm‐NPs) alongside semiconductor NPs,^[^ ^6^ ^]^ the topic of interest here. The advantage of this approach is that pm‐NPs can be fine‐tuned to absorb low‐intensity visible light (such as sunlight, as opposed to high‐intensity lasers) by controlling their size, shape and composition.^[^ ^7^ ^]^ By manipulating these parameters, it is possible to design nanostructures that can interact with the entire solar spectrum potentially. Thus, incorporating pm‐NPs into photocatalytic systems presents prospects for enhancing solar energy conversion.

However, such pm‐NP/semiconductor catalysts still perform poorly delivering low internal quantum efficiencies.^[^ ^8^, ^9^, ^10^ ^]^ Utilizing a computational framework, this perspective aims to shed light on the underlying mechanistic processes that lead to this problem and explore a potential solution in the form of modifying the pm‐NP/semiconductor interface by incorporating a non‐plasmonic metal in between. We briefly review the key plasmonic processes at play at such interfaces and discuss the solution strategy. We then present computational approaches and studies that can capture the plasmonic processes accurately and provide preliminary support to the solution strategy discussed. The discussion is primarily led through computational results while providing supporting experimental evidences wherever relevant. We conclude by providing an outlook on future computational and experimental research that needs to be carried out to further validate the proposed solution.

## 2 Plasmon Formation and Decay at pm‐NP/Semiconductor Interface

### 2.1 General Life Cycle

**Figure 1:**
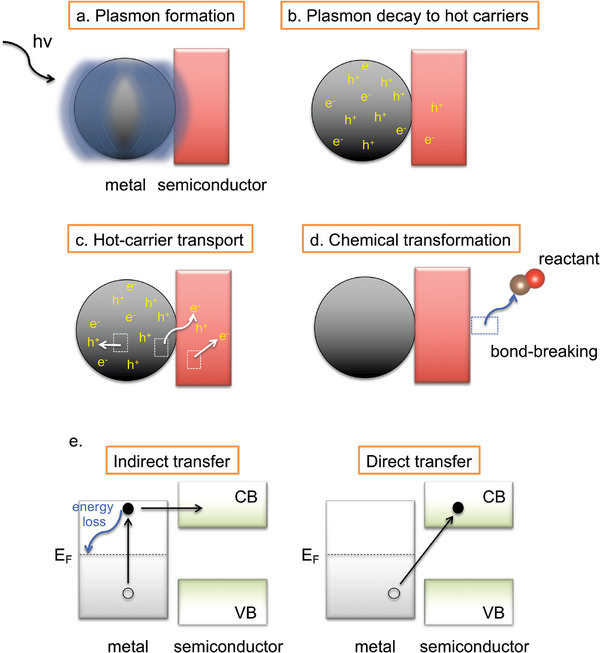
Plasmon formation and decay processes at pm‐NP/semiconductor interfaces. a–d) Schematic of the different plasmonic hot‐carrier processes at a pm‐NP/semiconductor interface. e) Schematic representation of the hot‐electron transfer processes from the metal to the semiconductor via indirect‐ and direct‐transfer processes. The energy loss associated with the indirect‐transfer process is also indicated. E_F_, CB, and VB represent the Fermi level, the conduction band and the valence band, respectively. Full and hollow circles denote electrons and holes, respectively. Not shown here is the carrier replenishment process. A similar scheme can be drawn for the hot‐hole transfer process.

The general life cycle and the associated steps of plasmon formation and decay at a pm‐NP/semiconductor interface is elaborated in **Figure** **1**a–d. The cycle starts with pm‐NPs strongly absorbing light owing to the localized surface plasmon resonance (LSPR) phenomenon (Figure 1a). LSPR is the coherent coupling of the incoming photons with the conduction electrons, which happens when the frequency of the former matches the natural frequency of the latter oscillating against the restoring force of positive nuclei.^[^ ^4^ ^]^

LSPR undergoes decay within a few femtoseconds (around 10 fs) through photon scattering into the far field or through the creation of single electron‐hole pair excitations within the pm‐NP (Figure 1b).^[^ ^7^ ^]^ The process of photon scattering, whose rate constant is proportional to the square of the particle's volume, dominates plasmon relaxation in relatively large particles of materials like Ag and Au (typically over 70 nm). Conversely, for smaller NPs (those under 20 nm), photon absorption followed by the formation of single electron–hole pairs, becomes the predominant process, which is crucial for driving chemical reactions.^[^ ^11^, ^12^ ^]^ The energy of the generated electron–hole pairs equals the energy of the incident photons.

When a pm‐NP is bundled up with a semiconductor, a Schottky junction is established. The electron‐hole pairs that are generated via plasmon decay in the metal can flow to the semiconductor (Figure 1b,c), which are expected to facilitate redox chemical transformations at the surface of the semiconductor by depositing their energy into the reactants (Figure 1d).^[^ ^13^ ^]^ The type of charge transfer primarily hinges on the band alignment at the pm‐NP/semiconductor interface. Generally, in an n‐type (p‐type) Schottky contact, hot electrons (hot holes) move from the pm‐NP to the semiconductor.^[^ ^14^ ^]^ More detailed descriptions of the different steps involved in the life cycle have been well‐documented in the literature.^[^ ^15^, ^16^ ^]^

### 2.2 Hot‐Carrier Transfer Mechanisms

The hot‐carrier transfer from the metal to the semiconductor can occur via two mechanisms (Figure 1e): (i) indirect transfer, where the hot electrons and holes are first produced in the metal and eventually get transferred to the semiconductor, (ii) direct transfer, where the surface plasmons interact directly with the energy states of the semiconductor, inducing direct carrier injection into these states.^[^ ^16^ ^]^ Regardless of the mechanism, these processes involve funneling photon energy into the semiconductor mediated by the metal.

The efficiency of transferring hot carriers to the semiconductor is known to be significantly higher in the direct‐transfer process compared to the indirect one.^[^ ^7^ ^]^ This is because, in the indirect process, there is a high probability of hot electrons and holes recombining within the pm‐NP before they can migrate into the semiconductor, resulting in the loss of photon energy as heat.^[^ ^17^ ^]^ Conversely, in the direct transfer process, where carriers are directly injected into the semiconductor through plasmon decay, they are already spatially separated, reducing the likelihood of recombination.

Although the direct‐transfer process helps improve charge‐transfer rates, it is often observed that the indirect‐transfer process is the dominant charge‐transfer pathway if the pm‐NP/semiconductor interface is not appropriately designed (or electronically coupled). Researchers have proposed initial solutions, such as directly coating the semiconductor onto plasmonic metal NPs to enhance interfacial area and electronic coupling,^[^ ^18^, ^19^, ^20^ ^]^ but recent work has shown that half of the charge‐carrier transfer still originates from the indirect‐transfer pathway.^[^ ^20^ ^]^ Taken together, these limitations contribute to the problem of poor quantum efficiencies observed in plasmonic devices.

## 3 A Potential Strategy to Improve Hot‐Carrier Transfer at pm‐NP/Semiconductor Interface

**Figure 2:**
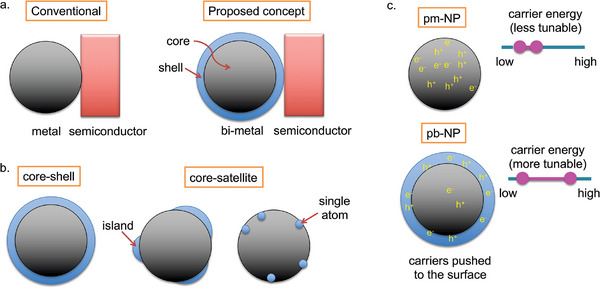
a) Schematic comparing the conventional pm‐NP/semiconductor configuration to the proposed pb‐NP/semiconductor design (specifically bimetallic core‐shell; see main text for the definitions of pm‐ and pb‐NP), which allows for a thin layer of a non‐plasmonic layer to be sandwiched between the plasmonic core and the semiconductor. b) Schematic comparing core‐shell and core‐satellite pb‐NP structures. The former involves covering the entire core with a non‐plasmonic metal, while the latter involves depositing different sizes of the non‐plasmonic metal on the core ranging from islands down to the single‐atom level. c) Schematic highlighting the benefits of pb‐NP over pm‐NP (the semiconductor is omitted for clarity). The former is expected to generate a higher quantity of electron–hole pairs at the surface of the particle compared to the latter. Depending on the shell metal composition, the hot‐carrier energy could also be tuned in pb‐NP.

Various groups have attempted to improve the efficiency of hot‐carrier transfer. Some of the strategies include altering the size of the NPs,^[^ ^21^ ^]^ epitaxial growth of semiconductor on the noble metal,^[^ ^22^ ^]^ NP doping,^[^ ^23^ ^]^ introduction of charge‐conducting nanomaterials such as reduced graphene oxide,^[^ ^24^ ^]^ among others. It is our view that the problem discussed above could also be potentially addressed by tailoring the pm‐NP/ semiconductor interface, which has been relatively underexplored. Specifically, drawing inspiration from the multicomponent plasmonic catalysis concept,^[^ ^15^, ^25^ ^]^ we postulate that introducing a thin layer of non‐plasmonic metal between the plasmonic‐metal and the semiconductor could help improve hot‐carrier migration into the semiconductor. One way to realize this experimentally is to make core‐shell bimetallic NPs, where a plasmonic metal such as Ag forms the core, while a non‐plasmonic metal such as Pt, forms a shell surrounding the core, and couple these particles with the semiconductor (see **Figure** **2**a comparing the conventional approach and the proposed design). Alternatively, rather than introducing an entire shell around the core, one could make core‐satellite structures, where different sizes of non‐plasmonic metal islands (down to the single‐atom level) can be introduced on top of the core plasmonic NP, which are then interfaced with the semiconductor (Figure 2b). Hereafter, we use the term pb‐NP (bimetallic NP with a plasmonic core) to encompass both core‐shell and core‐satellite structures to maintain brevity. Through strategic pb‐NP design, we expect that the flow of hot carriers across pb‐NP/semiconductor interfaces could be enhanced, as compared to their flow across pm‐NP/semiconductor interfaces, thus ensuring they enter the semiconductor before recombination occurs within the metal.

It should be noted that pb‐NPs have been explored previously in the so‐called multicomponent plasmonic catalysis, where the aim is to drive the chemical reactions directly on metallic NPs without the use of semiconductor NPs.^[^ ^15^ ^]^ There, the motivation to design pb‐NPs^[^ ^25^ ^]^ or antenna‐reactor‐type NPs^[^ ^26^ ^]^ instead of pm‐NPs stems from two requirements: (1) the need to reroute plasmon‐induced electron‐hole pair generation process to the surface (as opposed to the bulk) of the NP so that they can more easily reach the molecular orbitals and trigger chemical reactions, and (2) the fact that plasmonic metals like Ag, Au, and Cu may not be suitable catalysts for the chosen reaction. The pb‐NPs act as antenna‐reactor systems,^[^ ^15^, ^25^ ^]^ where the core helps absorb light,^[^ ^27^ ^]^ while the shell metal is usually a catalyst for a specific reaction.^[^ ^28^, ^29^, ^30^ ^]^ For example, Linic and co‐workers^[^ ^25^ ^]^ used this concept and synthesized Ag nanocubes (∼75 nm in length) surrounded by a very thin layer of Pt (∼1 nm). They used this structure to preferentially oxidize CO in a reactor with abundant H_2_. Such a pb‐NP system profited from the excellent absorption properties of Ag and the good catalytic properties of Pt along with improved hot‐carrier generation at the surface.

One constraint with this approach is that the non‐plasmonic metal chosen should satisfy both the aforementioned requirements, i.e., it should not only be a good catalyst for the reaction at hand, but also have the right electronic structure that provides hot‐carriers with sufficiently high energy to catalyze the reaction. We hypothesize that in a pb‐NP/semiconductor hybrid catalyst, these two functions could be decoupled. This is because, in the proposed concept (Figure 2a), the semiconductor performs the role of the catalyst, while the shell metal is not intended to act as a catalyst, but would solely serve to enhance hot‐carrier separation and transport from the plasmonic‐metal core to the semiconductor. We anticipate this enhancement due to the similarly intriguing properties the shell demonstrated in the case of multicomponent plasmonic catalysis (Figure 2c). Alongside shifting the spatial position of hot‐carrier generation to the surface of the pb‐NP, this approach offers scope to potentially tune the energies of the generated hot electrons and holes by altering the composition of the non‐plasmonic metal. For instance, non‐plasmonic materials such as Pt, Pd, and Ru, have a different electronic structure (such as partially filled *d*‐bands bringing them closer to the Fermi level) compared to plasmonic materials like Ag, thereby presenting exciting possibilities for tailoring the properties of pb‐NPs.

## 4 Probing Plasmonic Hot‐Carrier Processes Using Computational Modeling

Probing the hot‐carrier generation and transfer processes at the pm‐NP and pb‐NP/semiconductor interfaces would be necessary to understand the benefits of pb‐NPs over pm‐NPs. Because these processes occur on ultrafast timescales (1 fs – 1 ps),^[^ ^31^ ^]^ characterizing the relevant interfaces via experiments would be complicated and time‐consuming. Additionally, due to the two different types of charge‐transfer processes taking place in tandem (direct and indirect), isolating one individual mechanism from the other in experiments is not an easy task.^[^ ^32^ ^]^ This provides the rationale to employ computational approaches to tackle this issue. There is also a strong need for *ab initio* computational approaches in particular because the intended pm‐ and pb‐NP/semiconductor assemblies are typically nano‐sized structures (1 – 100 nm); as such, the atomic granularity and quantum effects such as charge‐density spillover, non‐local screening of electrons, tunelling transport, energy‐level quantization and quantum confinement among others become critical to accurately describe plasmon formation and hot‐carrier generation/separation processes.^[^ ^33^ ^]^ Furthermore, the precise disposition of the atoms at the interface will play a crucial role and strongly influence the nature of the plasmon formed there and the hot‐carrier transfer rates across them. Hence, being able to capture these features is a necessary requirement for predictive simulations.

A logical step would be to invoke quantum‐mechanical methods. Here again, several approaches with different advantages/disadvantages are available. For instance, the so‐called jellium models have been explored,^[^ ^33^, ^34^, ^35^, ^36^ ^]^ but they have some limitations. For example, they cannot describe the effects of the atomic structure and the impact of the *d*‐band is not accounted for (which is crucial for addressing *d*‐band plasmonic metals such as Ag, Au and Cu for instance). Besides this, there are many density functional theory (DFT) codes such as VASP,^[^ ^37^ ^]^ Quantum Espresso,^[^ ^38^ ^]^ NWChem,^[^ ^39^ ^]^ ABINIT,^[^ ^40^ ^]^ JDFTx,^[^ ^41^, ^42^ ^]^ etc. that capture the electronic band structures of molecules and solids, and utilize additional modules to compute the photon‐electron coupling at different levels of accuracy. For example, in VASP, one can compute the frequency‐dependent dielectric response functions such as the absorption spectra within the random phase approximation (RPA), namely the Fermi's Golden Rule applied to the Kohn–Sham (KS) states.^[^ ^43^ ^]^ This approach accounts for only a part of electron–electron interactions, i.e. the so‐called Coulomb kernel, but neglects the so‐called exchange‐correlation kernel, *f*_xc_ (see ref. [44] for more information). One could combine classical description of a plasmon field with a quantum‐mechanical treatment of the electrons/holes.^[^ ^45^, ^46^, ^47^ ^]^

As the current state‐of‐the‐art, researchers have begun to embrace time‐dependant DFT (TDDFT),^[^ ^48^ ^]^ a purely quantum‐mechanical method that provides a parameter‐free quantitative description of plasmon‐induced processes. The growing interest in utilizing this method stems from developments in efficient numerical algorithms in the past years that have enabled TDDFT modeling and analysis of NPs (or systems) up to nanometers in dimensions.^[^ ^49^, ^50^, ^51^, ^52^, ^53^, ^54^, ^55^, ^56^, ^57^, ^58^ ^]^ In particular, the real‐time TDDFT (RT‐TDDFT) approach (as implemented in the GPAW code^[^ ^59^ ^]^ for example) has been gaining popularity (as opposed to the Casida approach) because it scales favorably (linearly) with system size and can even account for non‐linear laser pulses.

In addition to robust algorithms, the development of post‐processing tools to analyze the output of RT‐TDDFT simulations has led to more detailed insights of the plasmonic hot‐carrier processes. The key feature is that the spectral, spatial and temporal distributions of the hot carriers can be quantitatively evaluated.^[^ ^53^, ^54^ ^]^ Hence, it is our view that RT‐TDDFT is ideally suited for researchers to take advantage of these well‐developed modules to explore plasmonic hot‐carrier processes at pm‐ and pb‐NP/semiconductor interfaces. It would be informative to study plasmonic characteristics in pm‐NP, pm‐NP/semiconductor, pb‐NP and pb‐NP/semiconductor systems in order to facilitate a step‐by‐step understanding. The following sections detail the current progress made toward this endeavor, how these current results suggest pb‐NP/semiconductor interfaces could perform better, followed by our perspectives on the future work one could pursue to develop a more complete picture.

### 4.1 Modeling of pm‐NPs

**Figure 3:**
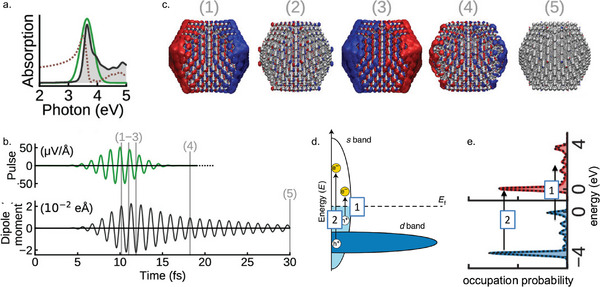
Plasmonic hot‐carrier characteristics in pm‐NPs from RT‐TDDFT simulations. a) Photoabsorption spectrum of the Ag_561_ pm‐NP (shaded black line) and the intensity profile of the Gaussian light pulse (green line). The real part of the polarizability is shown as a dotted line. b) The light pulse striking the pm‐NP in time domain (above), and the time‐dependent dipole‐moment response of the pm‐NP (below). c) Charge‐density oscillations in the pm‐NP at different time stamps (red and blue isosurfaces indicate electron and hole density, respectively. d) Schematic of the density of states of a plasmonic metal such as Ag, with an extended *s* band and a *d* band below the Fermi level. The expected intraband and interband excitations are indicated by the arrows 1 and 2, respectively. Reproduced with permission.^[^ ^15^ ^]^ Copyright 2018, Nature Publishing Group. e) Electron‐ and hole‐state occupation probabilities (hot‐carrier distribution) at time stamp 4, where plasmon has mostly decayed. The intraband and interband excitations indicated match those shown in the (d). a–c,e) Reproduced with permission.^[^ ^53^ ^]^ Copyright 2020, American Chemical Society.

As a first step, RT‐TDDFT captures the physics of plasmon excitation and decay to hot carriers in pm‐NPs accurately. In a study by Rossi and co‐workers,^[^ ^53^ ^]^ the computed photoabsorption spectrum revealed a clear LSPR peak around 3.6 eV (black line in **Figure** **3**a) for a model Ag_561_ pm‐NP. When the LSPR was excited using a monochromatic Gaussian light pulse (green line in Figure 3a), whose frequency is tuned to the plasmon resonance, a strong dipole‐moment response is observed because of the interaction of light with the electrons (Figure 3b). Additionally, the corresponding charge‐density maps reveal surface‐to‐surface cumulative electron‐density sloshing, which is consistent with the classical picture of a LSPR (Figure 3c, time stamps 1–3). Upon further analysis, this charge density is found to be composed of delocalized valence electrons, with a background screening due to virtual excitations from the d‐band. Interestingly, with time, the plasmon decay to hot carriers is also captured, wherein it is seen that the electron oscillation starts to lose its collective behavior and becomes more scattered (Figure 3c, time stamp 4), with a corresponding decay observed in the dipole moment. This decay is attributed to a dephasing process called Landau damping.

The computed hot‐electron and hot‐hole distributions (at time stamp 4, Figure 3d,e) following plasmon decay capture the two types of electronic transitions that are expected to occur in such pm‐NPs:^[^ ^15^, ^46^ ^]^ (1) the excitations from filled *s* states below the Fermi level to empty *s* states above the Fermi level, which are known as intraband excitations, and (2) the excitations from filled *d* states below the Fermi level to empty *s* states above the Fermi level, which are known as interband excitations. The probability of the interband transitions is observed to higher than that of the intraband transitions given the relatively high density of *d* states compared to the density of *s* states below the Fermi level.

The study also captures the sensitivity of hot‐carrier generation to atomic scale details and the resulting strong spatial variation. Especially, the corner‐ and edge‐atom sites of the pm‐NP, which are of relevance in catalysis, exhibit enhanced hot‐carrier generation in comparison to the bulk. The prevalence of hot electrons on such lower‐coordinated surface sites already seems to suggest improved carrier transport to an adsorbed molecule or a semiconductor.

### 4.2 Modeling of pm‐NP/Semiconductor Interfaces

**Figure 4:**
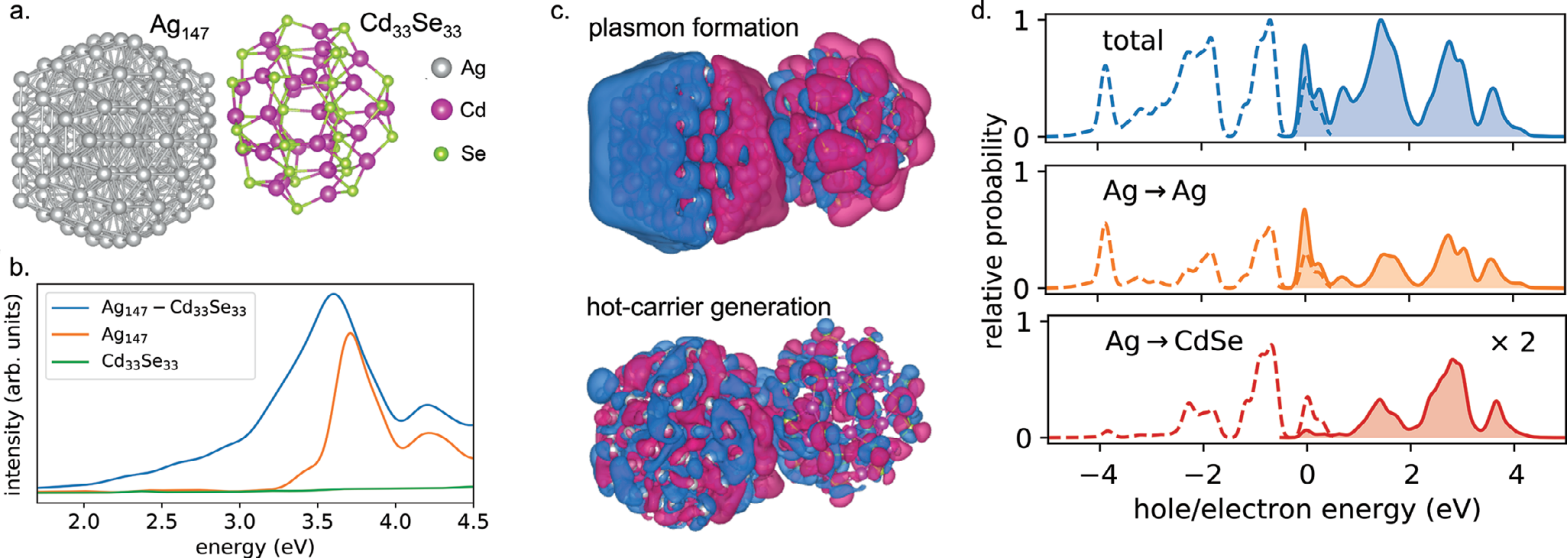
Plasmonic hot‐carrier characteristics in a pm‐NP/semiconductor assembly from RT‐TDDFT simulations. a) Ag_147_‐Cd_33_Se_33_ structural model used in the simulation. b) Photoabsorption spectra of Ag_147_, Cd_33_Se_33_ and Ag_147_‐Cd_33_Se_33_ structures. pm‐NP (shaded black line) and the intensity profile of the Gaussian light pulse (green line). c) Charge‐density oscillations revealing plasmon formation and subsequent hot‐carrier generation. Color code is similar to the one used on Figure 3c. d) The computed total and partial hot‐electron and hot‐hole distributions for different transitions. Not shown are the CdSe→Ag and CdSe→CdSe transitions. The dotted line in the total panel refers to the joint‐density of states. Reproduced with permission.^[^ ^54^ ^]^ Copyright 2019, American Chemical Society.

As a following step, some of us have used the RT‐TDDFT simulations to understand and quantify plasmonic hot‐carrier processes at pm‐NP/semiconductor interfaces.^[^ ^54^ ^]^ Using a model Ag_147_‐Cd_33_Se_33_ interface (**Figure** **4**a), these calculations showed that the plasmon peak of Ag_147_ around 3.7 eV is reduced and broadened upon bonding with the Cd_33_Se_33_ semiconductor due to additional plasmon‐decay channels that are introduced (Figure 4b). When the LSPR is excited with a Gaussian light pulse, similar to the study discussed above, cumulative charge‐density sloshing emerges, which decays by exciting single electron‐hole transitions leading to hot carriers (Figure 4c).

This study showed that RT‐TDDFT could distinguish between indirect‐ and direct‐transfer processes. It was noted that while the hot carriers are excited within the pm‐NP, they are also excited across the interface, which are shown in Figure 4d as Ag→Ag and Ag→CdSe excitations, respectively. The former is expected to lead to hot electrons and holes that could either recombine or get transported across the interface into the semiconductor given enough time following electron–electron and electron–phonon scattering processes (indirect transfer). However, the physics of these phenomena are not fully included in the description, and hence these steps could not be observed. On the other hand, the carriers excited across the interface immediately upon plasmon decay form the direct‐transfer component. The analysis estimated that the direct injection of both electrons and holes into CdSe happens within 10 fs of plasmon decay and with probabilities of about 20% each. It was inferred that the strong hybridization between the metal and the semiconductor electronic states plays a key role in facilitating such substantial charge‐injection probabilities.

These simulations are found to be consistent with experimental reports characterizing plasmonic hot‐carrier transfer at pm‐NP/semiconductor interfaces. For example, one of the first reports in the field by Lian and co‐workers revealed the plasmon‐induced interfacial charge‐transfer transition (PICTT) (or the direct‐transfer mechanism) at pm‐NP/semiconductor junctions using femtosecond pump‐probe spectroscopy.^[^ ^60^ ^]^ Demonstrated in CdSe nanorods with plasmonic Au tips, PICTT involves the direct excitation of electrons from the metal to a closely coupled acceptor, achieving a high quantum efficiency (>24%) that remains consistent across various excitation photon energies and is polarization‐dependent. Another report by Petek and co‐workers used ultrafast two‐photon photoemission spectroscopy to demonstrate the direct‐transfer pathway across a Ag/TiO_2_ interface.^[^ ^31^ ^]^ Time‐resolved measurements revealed plasmon decay and direct excitations of the hot electrons between Ag and TiO_2_ within 10 fs of plasmon formation, in line with the RT‐TDDFT study.

### 4.3 Modeling of pb‐NPs

**Figure 5:**
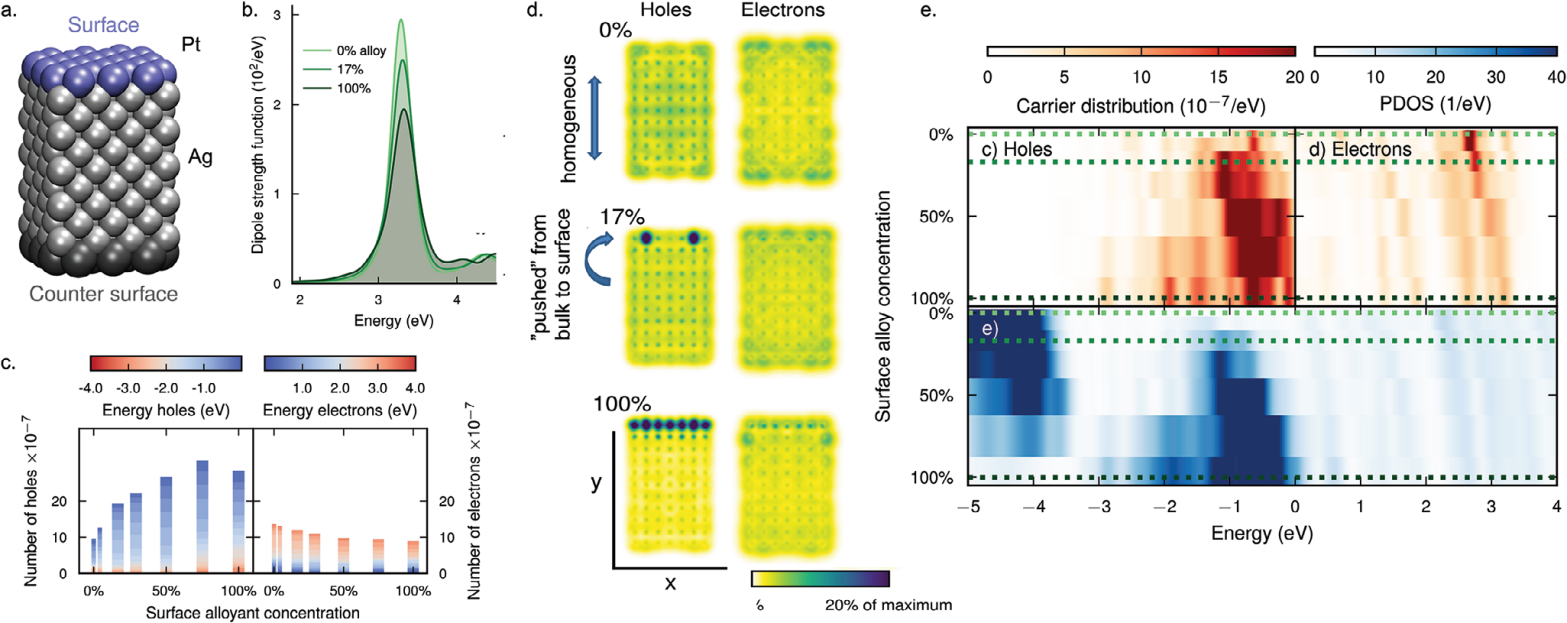
Plasmonic hot‐carrier characteristics in a pb‐NP from RT‐TDDFT simulations. a) Structural model used to model a pb‐NP system, where the framework is a 269‐atom Ag nanorod with one of the Ag surface layers being replaced by Pt. The concentration of Pt in the surface layer is varied in the study. b) The calculated photoabsorption spectra of pure Ag and Ag with the presence of different concentrations of Pt in the surface layer. c) The number of hot carriers generated after plasmon decay as a function of the Pt concentration in the surface layer. The corresponding hot‐carrier energies are also shown. d) The hot‐carrier spatial distribution maps showing that the hot holes tend to accumulate at the surface in expense of the bulk as the Pt concentration in the surface layer increases. e) Hot‐carrier distribution (top panel) and the corresponding projected density of states (PDOS, bottom panel) plots as a function of the Pt concentration in the surface layer. Reproduced with permission.^[^ ^61^ ^]^ Copyright 2024, American Chemical Society.

Having demonstrated RT‐TDDFT to be an excellent tool to describe plasmon physics in both pm‐NP and pm‐NP/semiconductor systems, we made headway in understanding the same in pb‐NPs, and have recently investigated the impact of having a non‐plasmonic shell on hot‐carrier formation in a model Ag_269_ cuboidal NP.^[^ ^61^ ^]^ A comparison is drawn between pure Ag and Ag‐Pt pb‐NPs, where substitutions were made in the top surface with varying amounts of Pt (**Figure** **5**a). Because we are studying computationally tractable, relatively small NPs, covering the entire Ag NP with Pt will strongly dampen the plasmon mode, thus making it inconsequential to study the underlying physics of plasmonic hot‐carrier processes. Nevertheless, the results obtained from the configurations studied can shed light on experimentally relevant pb‐NP structures qualitatively, if not quantitatively.

Both Ag and Ag‐Pt particles studied exhibit a LSPR at 3.3 eV, attributed to excitation along their long axis (Figure 5b). The LSP peak decreases, and its width increases with increasing Pt concentration, aligning with experimental findings.^[^ ^25^ ^]^ This is expected because the imaginary part of the dielectric function of Pt is higher than that of Ag, which serves to attenuate plasmon formation and introduce additional plasmon decay channels.^[^ ^7^ ^]^

Once the LSP is excited in these systems using an appropriate laser pulse, it is found that the plasmon formed decays by forming single electron–hole pairs within the system—similar to the case of pure Ag NPs. However, the presence of the chemically‐bonded Pt introduces additional electron–hole excitation pathways, thus altering the spatial distribution of hot carriers in such pb‐NPs compared to their pure counterparts. In this specific case of Ag‐Pt, the number of holes generated at the surface increases significantly with increasing Pt concentration, which makes these systems interesting for enhancing oxidation reactions (Figure 5c). On the other hand, the number of electrons at the surface is found to decrease slightly. This can be further understood from the hot‐carrier distribution maps (Figure 5d). These show homogeneous distribution of hot holes within pure Ag. However, upon introducing the Pt shell, the hot holes are “pushed” to the surface from the bulk, thereby tuning their spatial distribution. For the case of hot electrons, this effect is seen to be minimal. It should be noted that previous electrodynamic simulations have also shown that photon energy absorption occurs predominantly in the shell, examining the specific case of Ag‐Pt.^[^ ^25^ ^]^ However, since these are classical simulations, finer details regarding how the electronic structure participates to enable stronger absorption cannot be probed.

The projected density of states (PDOS) plots in the surface layer provide insights into available surface states and rationalizes the observed changes in the spatial distribution of holes (Figure 5e). In pure Ag, the PDOS comprises Ag *sp* states above ∼ −4 eV, along with *d* states far below the Fermi level. As such, the *d* states do not contribute to the hole distribution, being farther from the Fermi level than the energy provided by the resonant laser pulse (3.3 eV). Upon substituting Pt into the surface, Pt *d* states emerge between −1.5 and −0.5 eV, concurrently with a reduction in the number of Ag *d* states (around −4 eV). This gradual shift from an Ag‐like *d* band to a Pt‐like *d* band with increasing concentration aligns with the increased hole formation after LSP excitation.

**Figure 6:**
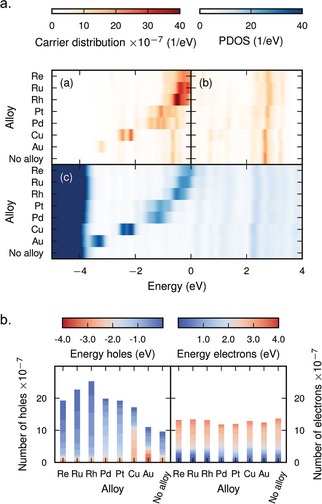
Tuning the hot‐carrier distribution through pb‐NP chemistry. a) Hot‐carrier distribution (top panel) and the corresponding partial density of states (bottom panel) plots with different surface (or shell) compositions. b) The number of hot carriers generated after plasmon decay as a function of different surface compositions. Reproduced with permission.^[^ ^61^ ^]^ Copyright 2024, American Chemical Society.

The influence of shell‐metal composition on hot‐carrier distribution was analyzed in the same study using seven *d*‐block elements. The PDOS in the surface layer (**Figure** **6**a) reveals that the *d* states shift closer to the Fermi level as an element further to the left in the periodic table is chosen as the shell, indicating control over the electronic structure of the NP surface. Similar to the Pt case discussed above, the resulting hole distribution follows the surface PDOS closely, thus allowing for the tuning of the hot‐hole distribution by controlling the shell‐metal composition. This tunability holds significance as the range of hot‐hole energies spans from −4 to 0 eV. Additionally, the number of hot holes can also be varied depending on the surface composition (Figure 6b), indicating further control over hot‐carrier properties in pb‐NPs.

The study mentioned above does not take the semiconductor into account; however, if a semiconductor is coupled to the pb‐NP, the discussed results are largely expected to remain applicable, aside from the introduction of additional plasmon decay channels caused by electronic coupling. While a first‐principles study directly comparing the charge‐transfer efficiencies of pm‐NP and pb‐NP/semiconductor systems is currently unavailable, developing a simplified process model could provide valuable insights into the advantages offered by pb‐NP over pm‐NP in a more quantitative way. Below, we present the framework of such a model and emphasize its key features.

### 4.4 Understanding Charge Transfer at Metal/Semiconductor Interfaces Using a Process Model

Based on the work by White and Catchpole,^[^ ^62^ ^]^ one can model the charge transfer (or current density response) across the metal/semiconductor junction using the equation:

(1) $$j_{res}=j_{sc}-A^{*}T^{2}e^{-\Phi_{\beta }/kT}\left(e^{V/kT}-1\right)$$

where, *j*_*sc*_ is the short‐circuit photocurrent density, and the second term on the right is the reverse current based on thermionic emission over the Schottky barrier, Φ_β_. In this second term, *A* is the modified Richardson constant, *k* is the Boltzmann constant, *T* is the temperature, and *V* is the voltage across the plasmonic device. We could write the total short‐circuit current density as:

(2) $$j_{sc}=j_{sc}^{it}+j_{sc}^{dt}$$

where, $j_{sc}^{it}$ and $j_{sc}^{dt}$ are the contributions of the indirect and direct transfer processes, respectively. We could further write expressions for $j_{sc}^{it}$ and $j_{sc}^{dt}$ as follows:

(3) $$j_{sc}^{it}=q\int \varphi \left(\lambda \right)\eta_{abs}\left(\lambda \right)\left(1-p^{dt}\left(\lambda \right)\right)\eta_{et}^{it}\left(\lambda \right)d\lambda$$

(4) $$j_{sc}^{dt}=q\int \varphi \left(\lambda \right)\eta_{abs}\left(\lambda \right)p^{dt}\left(\lambda \right)\eta_{et}^{dt}\left(\lambda \right)d\lambda$$

where, *q* is the charge on an electron, φ(λ) is the incident photon flux, η_*abs*_(λ) is the optical absorption in the metal NP, *p* ^*dt*^(λ) is the probability of the charge transfer occurring via the direct‐transfer process, and $\eta_{et}^{it}\left(\lambda \right)$ and $\eta_{et}^{dt}\left(\lambda \right)$ are the charge‐transfer efficiencies for indirect and direct processes, respectively. It should be noted that $\eta_{et}^{it}\left(\lambda \right)$ includes both transport through the pm‐NP (or pb‐NP) and emission over the Schottky barrier, while $\eta_{et}^{dt}\left(\lambda \right)$ includes direct emission over the Schottky barrier. As such, one would expect $\eta_{et}^{dt}\left(\lambda \right)$ to be higher than $\eta_{et}^{it}\left(\lambda \right)$. Taken together, one can write the full expression for *j*_*res*_ as:

(5) $$\begin{array}{ccc} & & j_{res}=q\int \varphi \left(\lambda \right)\eta_{abs}\left(\lambda \right)\left(1-p^{dt}\left(\lambda \right)\right)\eta_{et}^{it}\left(\lambda \right)d\lambda \\ & & +q\int \varphi \left(\lambda \right)\eta_{abs}\left(\lambda \right)p^{dt}\left(\lambda \right)\eta_{et}^{dt}\left(\lambda \right)d\lambda -A^{*}T^{2}e^{-\Phi_{\beta }/kT}\left(e^{V/kT}-1\right) \end{array}$$

This equation allows us to identify the parameters affected and understand the extent of their influence when a non‐plasmonic metal is introduced, compared to the scenario without it. We note that η_*abs*_(λ), *p* ^*dt*^(λ), Φ_β_, $\eta_{et}^{it}\left(\lambda \right)$ and $\eta_{et}^{dt}\left(\lambda \right)$ are expected to vary between the two cases considered. The optical absorption in the metal NP, η_*abs*_(λ), is expected to remain the same or decrease with the inclusion of the non‐plasmonic shell/crown due to plasmon screening. With advances in synthetic protocols of pb‐NPs, we expect good control over their optical properties by regulating NP shape and composition such that η_*abs*_(λ) could be minimally affected in experiments.

The probability of charge transfer occurring via direct transfer, *p* ^*dt*^(λ), could also be affected in pb‐NPs. For instance, we expect this to decrease if the shell/crown thickness is large and uncontrolled in experiments. However, we do believe that by keeping the thickness (or the amount) of the deposited non‐plasmonic metal shell/crown low and controlled, this factor could still be maintained or potentially even increased. This is because the non‐plasmonic metals tend to form stronger electronic bonds with the semiconductor atoms at the interface, which can increase the matrix element for the direct‐transfer process, and also, the plasmon sloshing is known to extend beyond the boundary of the core, thus being able to facilitate the direct process. This has been evidenced in a theoretical work by Meng and co‐workers, where the authors assessed the charge‐transfer processes at the Ag_19_Pt_1_‐CO_2_ interface and found higher charge transfer in the presence of Pt.^[^ ^63^ ^]^

The Schottky barrier, Φ_β_ value is also expected to be affected at the interface due to introduction of a third component. This depends on the composition of the non‐plasmonic metal introduced and is thus case‐specific. For instance, the introduction of a non‐plasmonic element that lowers Φ_β_ not only increases the short‐circuit current, but also the reverse current. Thus, the overall current density response can be assessed with Φ_β_ values derived for specific material systems.

We expect the charge‐transfer efficiency of the indirect process, $\eta_{et}^{it}\left(\lambda \right)$, to increase significantly in the pb‐NP case because the location of charge‐carrier generation via plasmon decay is pushed to the surface of the pb‐NP. As such, the charge transport improves because carriers have to travel much shorter distances to meet the semiconductor, thus lowering the probability of electron–hole recombination within the NP. Additionally, due to the presence of *d*‐states closer to the Fermi level in the case of a non‐plasmonic metal (say Pt) as opposed to a plasmonic metal (say Ag), more hotter electrons are expected to be generated and thus, more electrons with energy higher than Φ_β_ could be available to be emitted across the interface. This also increases the emission over the Schottky barrier and thus, the overall value of $\eta_{et}^{it}\left(\lambda \right)$. Since direct emission over the Schottky barrier is the only step in the direct process, we expect the charge‐transfer efficiency of the direct process, $\eta_{et}^{dt}\left(\lambda \right)$, to improve based on similar arguments presented above. Hence, $\eta_{et}^{dt}\left(\lambda \right)$ could potentially increase in the case of pb‐NPs.

It is important to recognize that, as discussed above, multiple factors are at play, and their interplay will ultimately determine the efficiency gains achievable with pb‐NPs compared to pm‐NPs. Therefore, a comprehensive parametric study in the future would be invaluable for providing a more detailed comparative analysis. Nevertheless, we hope that the discussion presented so far, emphasizing the potential benefits of incorporating a thin layer of non‐plasmonic metal between the plasmonic metal and the semiconductor, will inspire further experimental and theoretical research on the subject, some of which are outlined below.

## 5 Future Directions

### 5.1 Computational Research

**Figure 7:**
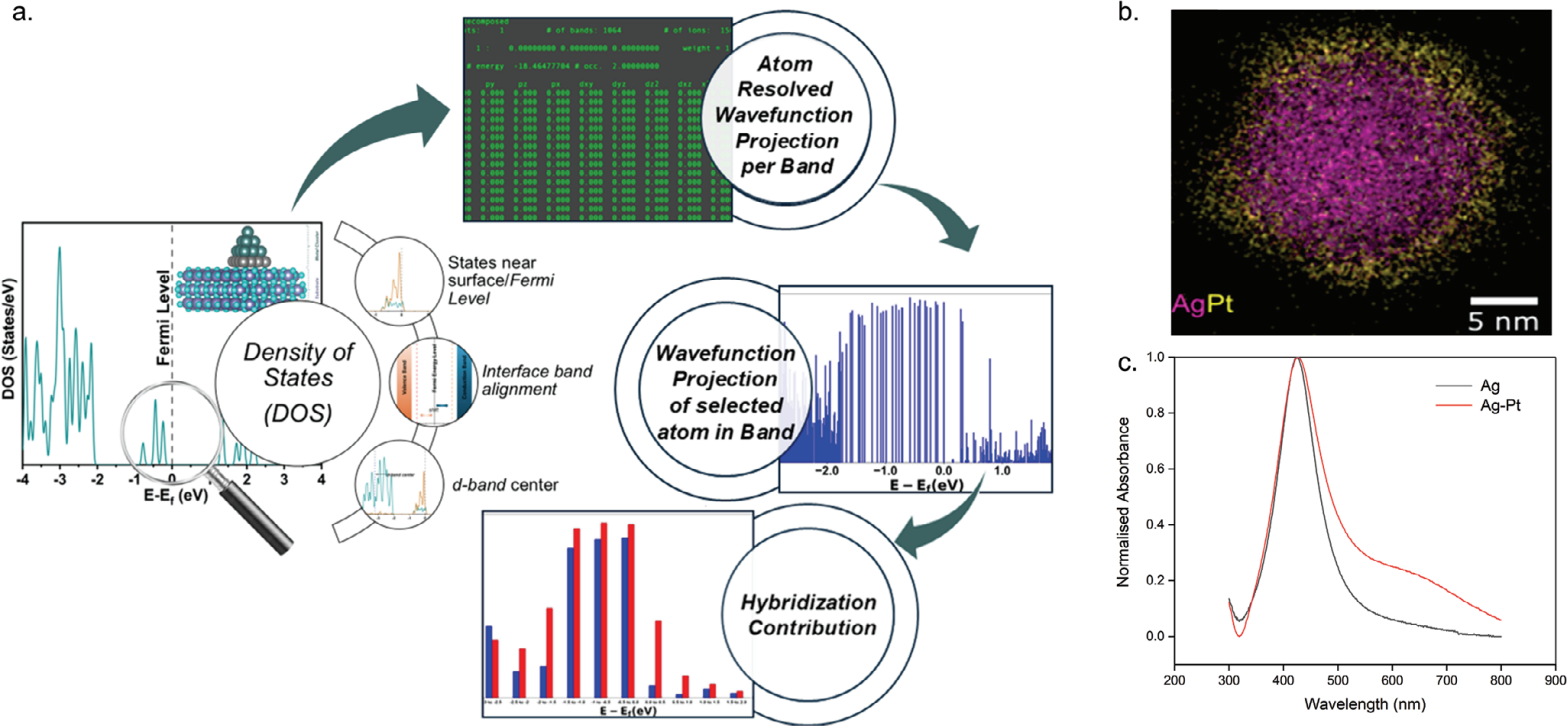
a) A schematic of the potential DFT analyses that could be performed prior to TDDFT simulations to understand the ground‐state properties of pm‐ and pb‐NPs, and the nature of their electronic bonding with semiconductors (see text for detailed description). b) Transmission Electron Microscopy elemental mapping of a single Ag‐Pt pb‐NP. Reproduced with permission.^[^ ^64^ ^]^ Copyright 2023, American Chemical Society. c) UV‐vis spectra of Ag‐Pt pb‐NPs grown using the recipe described in ref. [64] showing a plasmon peak around 420 nm.

The obvious next step in TDDFT modeling would be to include the semiconductor along with pb‐NPs. Before attempting TDDFT simulations, one could utilize standard DFT methods to analyse the differences between pm‐NP/ and pb‐NP/semiconductor interfaces. A schematic of the potential DFT analyses is shown in **Figure** **7**a. For these interface systems, two factors could be understood: (1) the electronic structure of individual pm‐NPs and pb‐NPs, and (2) interfacial properties when these NPs are integrated with the semiconductor. For instance, by comparing the PDOS of pm‐NPs and pb‐NPs, one can study how the electronic structure of the NP gets modified spatially (at the surface, for example) and spectrally (near the Fermi level, for example). Tracking and understanding these changes could already shed light on how these NPs bind to semiconductor. For example, as these NPs typically consist of *d*‐block elements, the shift in the *d*‐band center could be calculated and the corresponding *d*‐band theory could be invoked to reveal information about the nature of the bonding. Further analysis can also reveal the band alignment at the interface (n‐ or p‐type), which becomes important to assess the type of charge transfer from metal to the semiconductor.

It is also possible to get some insights on the ease of charge transfer at the interfaces by calculating orbital hybridization within DFT. Specifically, this could be done by analyzing the projected wavefunction data from the output files of a DFT calculation (for example, the PROCAR file in VASP) and calculating the overlap of electronic orbitals across defined energy ranges such as around the Fermi level, valence band, and conduction band. A systematic interval‐wise analysis could allow for a detailed understanding of how the non‐plasmonic‐shell metals influence hybridization compared to the case without the shell, thereby giving us an idea about charge‐transfer efficiency in the studied systems. Without having to resort to more complex TDDFT calculations, this approach could already assist in identifying optimal configurations that improve electronic coupling and improving the overall performance of plasmonic catalysts. It should be noted that these calculations purely provide insights on how charge carriers, if present in a particular orbital/band, could transport across the interface, but they do not compute the probability of charge‐carrier occupation in these orbitals/band via plasmon decay, for which one would need to resort to TDDFT.

Following these studies, thorough TDDFT simulations can help understand how the non‐plasmonic metal affects plasmon‐decay properties at the interface and more importantly, if/how it helps mediate indirect/direct charge transfers. These could include investigative TDDFT simulations exploring various shell compositions containing non‐plasmonic 4d and 5d metals such as Pd, Rh, Ru, Pt, etc., and their impact on charge transfer to the semiconductor. Parametric studies involving different shell thicknesses and different shell coverage on the core could be carried out. The key motivation would be to learn and quantitatively assess how plasmon energy is channeled across the interface.

Further methodology development is essential to fully describe hot‐carrier transfer processes. While TDDFT can account for plasmon formation and decay to hot carriers, it is well‐known that the resultant hot‐carrier distribution represents a system driven out‐of‐equilibrium, and this distribution moves toward a Fermi‐Dirac distribution following scattering processes. This represents an example requiring methodology development wherein the generated hot carriers will decay into multielectron excitations via electron‐electron scattering. In TDDFT, predominantly, adiabatic exchange‐correlation functionals are used, which do not capture this electron–electron scattering process. Hence, the development and utilization of non‐adiabatic exchange‐correlation functionals can overcome this issue.^[^ ^65^ ^]^ Furthermore, including various dissipative effects such as electron‐phonon scattering alongside electron–electron interactions is the key to model hot‐carrier separation/transport at interfaces, and describe processes such as the indirect‐transfer process quantitatively. There are some existing literature that have looked into how the indirect process occurs and how chemical bonds of a reactant are broken through transient hot‐carrier transfer by including electron‐phonon scattering,^[^ ^63^, ^66^, ^67^ ^]^ but they have been applied mostly to small systems, presenting limitations when dealing with modeling larger pm‐NPs and semiconductors. Specifically, it is our view that if the scattering physics is included within the linear combination of atomic orbitals (LCAO) formalism, for example in GPAW,^[^ ^68^ ^]^ it could present a possibility to model larger, more realistic systems. A more detailed treatment of methodology development can be found in the literature, for example in ref. [58]. Finally, we also note that a complete mathematical treatment of charge‐transfer process models, such as the one discussed earlier, would be beneficial to understand how different parameters should be controlled to optimize the charge‐transfer processes at pb‐NP/semiconductor interfaces.

### 5.2 Experimental Research

In parallel, experimental studies which involve synthesis and characterization of pm‐NPs, pb‐NPs and their interfaces with semiconductor will require attention. Concerning synthesis, it is crucial that the core and shell metals remain immiscible to preserve the integrity of the pb‐NP structure. Further, when designing pb‐NPs for plasmonic catalysis, it is important to maintain the LSPR peak of the NPs. Characterizations have shown that adding a non‐plasmonic material such as Pt can broaden or even diminish this plasmonic peak.^[^ ^25^, ^69^, ^70^, ^71^ ^]^ Therefore, the non‐plasmonic‐metal coating should be deposited in a way that minimizes this peak broadening. Thus, it is important to utilize protocols that render such systems.

For instance, some of us^[^ ^64^ ^]^ and others^[^ ^25^, ^72^ ^]^ have described approaches to synthesize Pt‐coated Ag NPs. Using the approach presented in ref. [64] core‐shell Ag‐Pt pb‐NPs with varying Pt island amounts/thickness can be obtained, and distinct immiscible, core and shell parts can be produced. An example transmission electron microscopy (TEM) elemental map showing a Pt island covering an Ag core, grown using the approach described above, is shown in Figure 7b. The corresponding optical absorption spectra of Ag and Ag‐Pt NPs (Figure 7c) reveal a clear LSPR peak around 420 nm. It is interesting to note that the plasmonic peak is preserved for Ag‐Pt NPs, indicating good control over Pt deposition without altering the morphology of the NPs significantly. The same plasmon peak is unsurprisingly wider, indicating additional channels for plasmon decay in Ag‐Pt core‐shell NPs, as highlighted in the previous section.

Researchers can make use of a variety of such synthetic protocols existing in the literature to make pm‐NPs, pb‐NPs and their semiconductor hybrids. For example, the synthesis of Ag pm‐NPs commonly utilizes chemical approaches, involving chemical reduction through diverse organic and inorganic reducing agents, as well as physical methods like evaporation‐condensation and laser ablation.^[^ ^73^, ^74^, ^75^, ^76^ ^]^ Additionally, electrochemical techniques,^[^ ^77^ ^]^ photochemical reduction^[^ ^78^, ^79^, ^80^, ^81^, ^82^, ^83^, ^84^, ^85^ ^]^ and radiolysis^[^ ^86^ ^]^ have also been explored.^[^ ^87^ ^]^ Diverse methods have also been reported for the synthesis of pb‐NPs, including chemical reduction,^[^ ^88^, ^89^ ^]^ microwave techniques,^[^ ^90^ ^]^ radiolytic processes,^[^ ^91^ ^]^ laser‐modified seed‐growth,^[^ ^92^ ^]^ laser‐induced alloying,^[^ ^92^, ^93^ ^]^ and electron beam deposition.^[^ ^94^ ^]^ Once the pm‐ and pb‐NPs are made, fabricating their composites with suitable semiconductors that have the ability to carry out redox reactions would be desirable. Deposition techniques for these composites include flame aerosol reactor,^[^ ^95^ ^]^ spray pyrolysis,^[^ ^95^ ^]^ electron beam evaporation system,^[^ ^96^ ^]^ dip‐coating,^[^ ^97^, ^98^ ^]^ spin‐coating,^[^ ^96^ ^]^ and drop‐casting.^[^ ^6^ ^]^

Once such hybrids are synthesized, pump‐probe spectroscopy presents a suitable way to study ultrafast processes in them, offering excellent temporal resolution,^[^ ^72^, ^99^ ^]^ potentially down to attoseconds, to track hot‐carrier transport and spectral distribution. While conducting these experiments is challenging, accurate execution can align with simulation results from TDDFT, providing detailed temporal, spectral, and potentially spatial insights into the investigated processes, thus aiding catalyst design. Advances in the development of attosecond pump‐probe spectroscopy have allowed for the resolution of sub‐femtosecond carrier dynamics.^[^ ^100^ ^]^ Here, a pump pulse can excite the plasmon. After a controlled time delay, an extreme ultraviolet attosecond probe pulse (covering the energy range from 20 to 60 eV) can be used to strike the sample and its absorbance/transmission could be measured.^[^ ^100^ ^]^ By monitoring the probe signal as a function of the time delay, it is possible to understand the changes in consequent photoabsorption and hot‐carrier‐injection events. There are certainly challenges associated with this method, such as the requirement of samples to be thin enough (∼100 nm) to transmit the probe pulse and to obtain noise‐free data. Because the ultrafast nature of the plasmons have not been fully understood at sub‐fs to fs timescales, the attosecond pump‐probe technique could prove to be yet another excellent, experimental tool to uncover novel physical phenomena.

Overall, as discussed, we believe that combining computational and experimental approaches can significantly accelerate progress in the field of plasmonic catalysis by providing complementary insights that refine and validate each other. Excellent examples in this direction exist in the plasmonics literature where computational techniques have provided rationale for unusual experimental observations for applications not only in catalysis,^[^ ^42^ ^]^ but also in other areas such as photodetection^[^ ^9^ ^]^ for instance. Leveraging the iterative feedback loop between computations and experiments can thus lead to a deeper understanding of the interfaces and their optimization.

## 6 Conclusion

In summary, recent first‐principles calculations indicate the potential for enhancing the transfer of hot carriers from plasmonic metal NPs to semiconductors by including a non‐plasmonic shell around the plasmonic‐metal core. This possibility is understood to stem from a shift in the spatial position of energetic electron‐hole pairs from within the core toward the surface of the NP. This is expected to help push the hot carriers across the interface before they can lose their energy via electron–electron and electron–phonon interactions. The inclusion of a non‐plasmonic metal also offers to tailor the energies of the generated hot carriers, as revealed from recent theoretical work. While first‐principles approaches such as TDDFT and experimental techniques such as the pump‐probe spectroscopy can already shed some light on plasmon decay to hot carriers and their spatio‐temporal distributions, they must be further developed and leveraged to further understand the plasmonic hot‐carrier processes, especially at bimetallic‐NP/semiconductor interfaces. Given the discussion presented in this perspective about the importance of including a non‐plasmonic‐shell metal, we hope that future studies will examine such interfaces and provide an in‐depth understanding in comparison with the unmodified interfaces. We expect this to impact not only the field of plasmonic catalysis, but also other areas such as photodetection, photovoltaics and photon upconversion for example, where plasmonic‐metal‐NP/semiconductor interfaces serve as active materials.

## Conflict of Interest

The authors declare no conflict of interest.
